# Correlates of different facets and components of beta diversity in stream organisms

**DOI:** 10.1007/s00442-019-04535-5

**Published:** 2019-10-17

**Authors:** Mariana Perez Rocha, Luis M. Bini, Mira Grönroos, Jan Hjort, Marja Lindholm, Satu-Maaria Karjalainen, Katri E. Tolonen, Jani Heino

**Affiliations:** 1 Geography Research Unit, University of Oulu, PO Box 3000, 90014 Oulu, Finland; 2 Freshwater Centre, Finnish Environment Institute, PO Box 413, 90014 Oulu, Finland; 3 Department of Ecology (ICB), Universidade Federal de Goiás, Goiânia, GO 74690-900 Brazil; 4 Faculty of Biological and Environmental Sciences Ecosystems and Environment Research, Niemenkatu 73, 15140 Lahti, Finland; 5 CAPES Foundation, Ministry of Education of Brazil, Brasília, 70040-020 DF Brazil

**Keywords:** Beta diversity, Biodiversity facets, Environmental gradients, Taxonomy, Traits

## Abstract

Recently, community ecology has emphasized the multi-facetted aspects of biological diversity by linking species traits and the environment. Here, we explored environmental correlates of taxonomically-based and traits-based compositional distances using a comprehensive data set of diatom and macroinvertebrate communities. We also explored the responses of different beta diversity components (i.e., overall beta diversity, turnover, and nestedness) of beta diversity facets (i.e., taxonomically and traits-based beta diversity) to environmental distances. Partial Mantel tests were used to test the relationships between beta diversity and environmental distance (while controlling for spatial distances). Taxonomically-based beta diversity varied much more than traits-based beta diversity, indicating strong functional convergence. We found that taxonomically-based beta diversity was largely driven by the turnover component. However, the nestedness component contributed more to overall traits-based beta diversity than the turnover component. Taxonomically-based beta diversity was significantly correlated with environmental distances for both diatoms and macroinvertebrates. Thus, we found support for the role of environmental filtering as a driver of community dissimilarities of rather different biological groups. However, the strength of these relationships between beta diversity and environmental distances varied depending on the biological group, facet, component, and the way which the environmental variables were selected to calculate the explanatory (distance) matrix. Our results indicated that both taxonomically and traits-based approaches are still needed to better understand patterns and mechanisms affecting the organization of biological communities in streams. This is because different facets of biological communities may be driven by different mechanisms.

**Electronic supplementary material:**

The online version of this article (10.1007/s00442-019-04535-5) contains supplementary material, which is available to authorized users.

## Introduction

Biodiversity refers to the variability of life on Earth, ranging from genetic variation to ecosystem diversity (Gaston 2000). Taxonomic species diversity, one of the most commonly studied facets of biodiversity, does not consider that species have different traits (Villéger et al. 2013). Therefore, this metric neglects the fact that different species have different ecological roles and functions (Petchey and Gaston 2006; Mouillot et al. 2013; Gagic et al. 2015), hindering the study of community assembly mechanisms (Swenson et al. 2011; Pavoine and Bonsall 2011). Recently, community ecology has used traits-based approaches to test different hypotheses regarding the mechanisms underlying biodiversity patterns (McGill et al. 2006; Edwards et al. 2013; Villéger et al. 2013; Van Looy et al. 2019). These different facets of biodiversity may be partly driven by different processes (Heino and Tolonen 2017; Gianuca et al. 2017). Thus, when investigating biodiversity patterns, the use of approaches based on taxonomic and trait information may provide complementary information about the processes shaping the organization of biological communities.

Biodiversity patterns can also be explored based on different components, i.e., alpha, beta, and gamma diversity (Whittaker 1960). Specifically, beta diversity informs us how community composition varies in space and time (Anderson et al. 2011). Beta diversity is a central quantity for both basic and applied ecology. Indeed, the search for factors determining variation in community composition has been a long-standing goal in basic community ecology (Tuomisto 2010; Tuomisto and Ruokolainen 2006). In an applied context, beta diversity is directly related to the complementarity principle (i.e., the selection of a set of areas which are complementary to each other in terms of representing, for example, a regional species pool), a fundamental tenet in spatial conservation prioritization (Bush et al. 2016). Recent developments have shown that beta diversity can be further decomposed into its turnover (i.e., species replacement between different sites) and nestedness (i.e., difference in the number of species between sites) components (Baselga 2010). Such a decomposition of total beta diversity can help us to understand the processes underlying the spatial variation in community composition. Also, this approach can be used on both taxonomically-based and traits-based data (Baselga 2012; Baselga and Leprieur 2015).

Community compositional dissimilarities may be related to spatial and environmental distances (Nekola and White 1999; Tuomisto et al. 2003; Soininen et al. 2007). For instance, species may differ in their environmental requirements and, thus, community compositional differences may be related to environmental differences between sites (Soininen et al. 2007; Heino and Soininen 2010). Also, dispersal barriers and dispersal limitation may increase community dissimilarity with increasing spatial distance (Cañedo-Argüelles et al. 2015; Kärnä et al. 2015). Previous studies from stream systems have found an increase in compositional dissimilarity with both environmental (Brown and Swan 2010; Cañedo-Argüelles et al. 2015) and spatial distances (Saito et al. 2015; Sarremejane et al. 2017). In general, stream studies have revealed that species composition changes mostly due to between-site environmental differences, whereas spatial distances are generally less important in accounting for compositional changes (Heino et al. 2015). However, most studies on stream organisms have focused on taxonomically-based compositional dissimilarities only (Cañedo-Argüelles et al. 2015; Kärnä et al. 2015). Consequently, it is still unclear how traits-based community dissimilarities are related to environmental differences (Lindholm et al. 2018; Tolonen et al. 2017).

In addition to adopting a multi-facetted approach, which includes taxonomic and trait compositions, and different beta diversity components (turnover and nestedness), our study focused on two groups of organisms: benthic macroinvertebrates and diatoms. We envisaged that including both groups which have different biological traits and ecosystem functions (Allan and Castillo 2007) in a single study would allow us to increase our understanding of stream biodiversity and community assembly in response to environmental variation. For example, a consistent correlation between beta diversity components and environmental distances for both groups of organisms, despite the differences in their ecosystem functions (e.g., detritus processing vs. autochthonous biomass production) and traits (e.g., dispersal ability and body size), would be a strong evidence for the role of environmental filtering.

Here, we first asked whether taxonomically and traits-based dissimilarity matrices are related to each other using two groups of organisms, macroinvertebrates and diatoms. A low relationship between these facets would suggest that they are likely to offer complementary information. Second, we tested for environmental correlates of different facets and components of beta diversity (while controlling for spatial distances). Because macroinvertebrates and diatoms groups are considered reliable ecological indicators, we predicted that both facets and components would respond significantly to environmental gradients (Heino and Tolonen 2017; Lindholm et al. 2018). However, because biological traits are likely to better represent the direct links between-species distributions and the environmental variation (Verberk et al. 2013), we expected to find stronger correlations among beta diversity components and environmental distances when these components were calculated using traits instead of species identities only. As indicated by recent meta-analyses (Alahuhta et al. 2017; Soininen et al. 2018), we also predicted that the turnover would be the largest component of total beta diversity.

## Methods

### Study area

The study area was located within a large subarctic river basin, the Tenojoki River (with a basin area of 16,386 km^2^), situated in northernmost Finland and Norway (Figure S1). Our biological and environmental data were collected from 54 streams in June 2012. Stream waters of the study area are characterized by circumneutral pH, whereas nutrient levels are indicative of ultra-oligotrophic conditions (Heino et al. 2003). Mostly, arctic–alpine vegetation characterizes the study area, with barren tundra at higher altitude and mountain birch (*Betula pubescens* ssp. *czerepanowii*) woodlands at lower altitude. Also, Scots pine (*Pinus sylvestris*) forests are present, mainly occurring in the southernmost parts of the drainage basin area. The bedrock includes common igneous rock types, such as granites, gneisses, gabbros, and diorites (Kärnä et al. 2015; Tolonen et al. 2016). The study area is currently under low anthropogenic pressure, and streams are in pristine or near-pristine conditions (Tolonen et al. 2017). The high natural variation in environmental conditions (e.g., depth, water flow, particle size, and channel width) among the streams provides an excellent opportunity to test our predictions. Specifically, natural environmental heterogeneity in our study area can be considered as high enough to allow investigating possible relationships between beta diversity and environmental distance (Kärnä et al. 2015). Testing the relationship between beta diversity and environmental distances in near-pristine systems is also important as a baseline for future bioassessment of more altered stream systems. For example, this pattern would indicate that different beta diversity components and facets are responsive to environmental variation, even considering the near-pristine nature of a system. This result, in turn, would reinforce their use in situations where environmental variation is mainly driven by anthropogenic activities. However, our study area, like high-latitude areas in general, is undergoing changes due to climate change (Wrona et al. 2013).

### Diatom and macroinvertebrate sampling and species traits

Diatom samples were taken at each of the 54 stream sites from 10 randomly collected stones at depths of 10–30 cm. Thus, we analyzed a total of 540 stones (54 streams × 10 stones). From each stone, an area of 25 cm^2^ (5 cm × 5 cm) was brushed to get a total brushed area of 250 cm^2^ for each stream (10 stones × 25 cm^2^) (i.e., creating 54 samples). These samples were pooled into a composite sample for each site and subsequently taken to the laboratory where they were treated using a strong acid solution (HNO_3_:H_2_SO_4_, 2:1) to oxidize frustules (SFS-EN 14407 2005). Subsamples of 500 valves per site were identified and counted under a differential interference contrast microscope with 1000 × magnification (Kelly et al. 1998). Most diatoms were identified to species level (c. 98%) and only few to genus level (c. 2%), totaling 116 taxa.

Diatoms were assigned to different morphological groups (i.e., low-profile, high-profile, motile, and planktic), cell-size classes (i.e., biovolume), and life-forms (i.e., colonial or non-colonial) (Rimet and Bouchez 2012). The low-profile group includes species of short stature and tightly attached to the substratum. The high-profile group comprises large species or those which tend to form colonies. The motile group consists of species able to move. The planktic group consists of species that possess features that help to resist sedimentation (e.g., *Aulacoseira alpigena* and *Diatoma tenuis*). The first three of the mentioned groups (i.e., low-profile, high-profile, and motile) are based on diatom growth morphology according to Passy’s (2007) approach, and the planktic group was suggested by Rimet and Bouchez (2012) to complement Passy’s (2007) classification. We used Rimet and Bouchez (2012) classes of biovolume (in µm^3^) as follows: 0–99 µm^3^ (S1), 100–299 µm^3^ (S2), 300–599 µm^3^ (S3), 600–1499 µm^3^ (S4), and ≥ 1500 µm^3^ (S5). Based on Round et al. (1990), we used the following classification of life-forms: non-colonial (i.e., solitary cells either attached or non-attached to substratum) and colonial (i.e., floating or attached diatoms).

Benthic macroinvertebrates were collected at each stream site by taking six 30 s kick-net subsamples (mesh size: 0.3 mm), which included most of the variation in depth, current velocity, particle size, and moss cover in the sampling area (ca. 100 m^2^). Thus, a 3-min composite sample was obtained for each of the 54 stream sites (Kärnä et al. 2015; Tolonen et al. 2017). In the field, samples were pooled (for each site) and preserved in ethanol. Most macroinvertebrates were identified to the species level (c. 77%), but early larval stages were identified to genus level (c. 23%), because some individuals did not show adequate morphological characteristics to allow identification to species level. We mostly used Nilsson (1996, 1997) and references therein to identify the macroinvertebrates. In total, we obtained a list of 106 taxa.

For macroinvertebrates, we used three groups of traits following Tolonen et al. (2016, 2017): functional feeding groups (FFGs), habit trait groups (HTGs), and body size categorizations (BS). FFGs are based on the mode of feeding and food type, and included filterers, gatherers, shredders, scrapers, and predators (Merritt and Cummins 1996; Tachet et al. 2010). Macroinvertebrates were assigned into FFGs according to Moog (2002), Merritt and Cummins (1996), and Tachet et al. (2010). HTGs yielded information about mobility and microhabitat use, and comprised burrowers, climbers, clingers, sprawlers, and swimmers. Macroinvertebrates were assigned to HTGs using information from Merritt and Cummins (1996) and Tachet et al. (2010). Each macroinvertebrate species was assigned to one FFG or HTG only, based on its most typical preferences in Moog’s (2002) or Tachet et al.’s (2010) classifications, complemented by information given in Merritt and Cummins (1996) and our own expert knowledge (Tolonen et al. 2016, 2017). The BS classes referred to maximum larval body length, and each taxon was assigned to one of six size categories: 0–0.25 cm (BS1), 0.25–0.5 cm (BS2), 0.5–1 cm (BS3), 1–2 cm (BS4), 2–4 cm (BS5), and 4–8 cm (BS6). Information about BS categories was obtained from personal communication with S. Dolédec (Université Lyon, France), J. Ilmonen (Metsähallitus, Finland), and L. Paasivirta (Salo, Finland).

### Environmental variables

Local environmental variables were measured immediately after taking the biological samples. Thirty random spots in a riffle site, for each of the 54 streams, were selected to obtain water current velocity (m s^−1^) and depth (cm). Stream width (m) was measured from five cross-channel transects. We visually estimated bottom particle size at 10 randomly selected 1 × 1 m quadrats applying a modified Wentworth scale (Wentworth 1922): sand (0.25–2 mm), gravel (2–19 mm), pebble (16–64 mm), cobble (64–256 mm), and boulder (256–1024 mm). Moss cover (%) was also assessed visually at the same quadrats. Shading (%) by riparian deciduous trees was visually estimated at the center of each sampling site. Other variables measured in the field included conductivity (μS cm^−1^) and pH using a YSI multiprobe field meter (model 556MPS; Yellow Springs Instruments, Yellow Springs, Ohio). In addition, water samples were frozen and subsequently analyzed in the laboratory for total nitrogen (µg L^−1^), color (Co–Pt), iron (µg L^−1^), and manganese (µg L^−1^), following the Finnish national standards (National Board of Waters and the Environment 1981). These environmental variables were chosen, because they have been found to be the most important ones in explaining variation in stream communities in northern regions (Tolonen et al. 2016; Lindholm et al. 2018). Total phosphorus was not measured due to its low values (less than 5 µg/L) occurring in this region (Heino et al. 2003) (see Supporting Information Table S1 for a statistical description of the local environmental conditions in our study area).

### Statistical analyses

By an additive partitioning of the total beta diversity (Sørensen) into turnover (Simpson) and nestedness-resultant components, we analyzed the variability in both taxonomically-based and traits-based beta diversity. Each facet and each component were analyzed separately for diatoms and macroinvertebrates using distance-based methods. All analyses were based on presence–absence data and were performed in the R environment (R Core Team 2018).

The analyses were implemented in two phases (Supporting Information Figures S2 and S3). The first phase comprised the generation of three dissimilarity matrices (i.e., total, turnover, and nestedness-resultant dissimilarity) based on either diatom or macroinvertebrate taxonomic data using the function “beta.pair” from the R package *betapart* (Baselga et al. 2013). Accordingly, Sørensen dissimilarity measures the total beta diversity, whereas Simpson coefficient measures spatial turnover, and nestedness measures the compositional differences caused by nested variation in species richness. Each of these three pairwise matrices was used in partial Mantel test. Partial Mantel tests (Smouse et al. 1986; Legendre and Legendre 2012) were run to test the relationships between compositional dissimilarities and environmental distances (see below), while controlling for spatial distances. Because the different components of beta diversity are given as pairwise distance matrices, the use of the Mantel approach is justifiable, despite some criticisms (e.g., Guillot and Rousset 2013). We used overland (Euclidean) distances as measure of spatial distances, because we sampled small-to-medium-sized tributary streams that were connected to each other by large rivers only. Thus, dispersal (via drift) between sites is highly unlikely, whereas aerial dispersal by wind (for diatoms) or by flying (for insects) should be more frequent. Also, overland and watercourse distances were highly correlated to each other (Mantel *r* = 0.901, *P* < 0.001). Partial Mantel tests were run using the function “mantel” from the R package “ecodist” (Goslee and Urban 2007).

In the second phase, three dissimilarity matrices (i.e., total, turnover, and nestedness-resultant dissimilarity) were produced as described above, but this time also utilizing trait data both for diatoms and macroinvertebrates. Thus, we obtained three functional dissimilarity matrices, following the approach proposed by Villéger et al. (2013). Before calculating these matrices, Gower distance (Gower 1971) was used to calculate between-species distances based on the trait data using the function “gowdis” from the R package *FD* (Laliberté et al. 2014). The Gower distance allows handling both qualitative and quantitative variables (Legendre and Legendre 2012). Then, we used these trait distances in Principal Coordinate Analysis (PCoA) to produce trait vectors (Villéger et al. 2013) using the function “pco” from the R package *labdsv*. Gower-trait distances were strongly correlated with between-species Euclidean distances based on the first two PCoA vectors when analyzing either diatom (*r*^2^ = 0.67) or macroinvertebrate (*r*^2^ = 0.75) data. These two trait vectors and the data on site-by-species matrix were then used to produce the three traits-based dissimilarity matrices (i.e., total, turnover, and nestedness-resultant dissimilarity) using the function “functional.beta.pair” from the R package *betapart* (Baselga et al. 2013). Thereafter, as described above for the taxonomically-based analyses, we proceeded with the same steps using partial Mantel tests.

### Explanatory (environmental) matrices

We used three approaches to select the environmental variables to be used to calculate the environmental distance matrices and, subsequently, in the partial Mantel tests (as described above). In all approaches, we calculated a standardized Euclidean distance matrix. First, we used all environmental variables (Table S1) to calculate an environmental distance matrix. Second, we selected a priori five environmental variables (for diatoms: nitrogen, color, pH, shading, and stream width; for macroinvertebrates: nitrogen, color, shading, current velocity, and stream width), which are known to affect the community composition and distributions of diatoms (e.g., Jyrkänkallio-Mikkola et al. 2017; Lindholm et al. 2018) and macroinvertebrates (e.g., Heino and de Mendoza 2016; Tolonen et al. 2016) in our study area and in other regions. These environmental variables affect stream diatom and macroinvertebrate communities, because they are related to a site’s trophic status and productivity (e.g., nutrients, shading), habitat area (e.g. stream width), and physiological stress to organisms (e.g., pH, current velocity). Third, we used the bio-env analysis (Clarke and Ainsworth 1993) to select the best set of environmental variables resulting in the highest possible correlation between biological dissimilarities and environmental distances (Table S2 and S3). The bio-env analysis works to produce the ‘best’ set of scaled environmental variables for the final environmental distance matrix. This method has been frequently used in different fields of ecology (Ellingsen and Gray 2002; Spear et al. 2005; Carson et al. 2007). Bio-env analysis was run using the function “bioenv” from the R package *vegan* (Oksanen et al. 2015). The third approach was considered important, because we do not know beforehand which environmental variables are important for different organismal groups, biodiversity facets, and components of beta diversity in a given drainage basin (Heino and Tolonen 2017). We acknowledge, however, that bio-env provides biased significance levels, because this analysis seeks to maximize the correlation between matrices (Oksanen et al. 2015). Thus, we used this method only to guide future research directions, where studies could test, using independent data sets, whether the lists of selected environmental variables are, indeed, related to the different components and facets of beta diversity.

## Results

### Taxonomically versus traits-based beta diversity

We found relatively weak, albeit significant (*P* < 0.05), correlations between taxonomically-based and traits-based beta diversity components (Supporting Information Table S4). For diatoms and macroinvertebrates, the highest Mantel correlations were found for the total beta diversity (*r* = 0.263 and *r* = 0.512, respectively), while the turnover components were the lowest (*r* = 0.231 and *r* = 0.366, respectively).

### Diatom taxonomically and traits-based beta diversity

Taxonomically-based dissimilarities among sites showed much more variation when compared with the traits-based dissimilarities (Table 1). Taxonomically-based diatom beta diversity (mean total beta diversity = 0.502) was mainly due to the turnover component (mean turnover component = 0.414), with a negligible contribution of nestedness (mean = 0.087). For traits-based beta diversity, mean values for the three components (i.e., total, turnover, and nestedness-resultant) were very low (mean values = 0.056, 0.014, and 0.041, respectively) in which the nestedness component contributed more to overall traits-based beta diversity than the turnover component (Table 1).

**Table 1:** Statistical descriptions of pairwise dissimilarities between sites for diatom and macroinvertebrate communities (i.e., taxonomically-based and traits-based beta diversity) and their components (i.e., total, turnover, and nestedness-resultant)

	Total	Turnover	Nestedness
*Diatoms*			
Taxonomically-based beta diversity			
Min	0.234	0.062	0
Max	0.804	0.800	0.378
Mean	0.502	0.414	0.087
SD	0.093	0.112	0.071
Traits-based beta diversity			
Min	0	0	0
Max	0.171	0.131	0.151
Mean	0.056	0.014	0.041
SD	0.039	0.021	0.035
*Macroinvertebrates*			
Taxonomically-based beta diversity			
Min	0.200	0.045	0
Max	0.809	0.772	0.455
Mean	0.481	0.387	0.093
SD	0.113	0.133	0.078
Traits-based beta diversity			
Min	0	0	0
Max	0.270	0.184	0.203
Mean	0.087	0.032	0.048
SD	0.051	0.036	0.040

Partial Mantel tests based on all variables showed that the environmental distances (after controlling for spatial distances) were significantly correlated with taxonomically and traits-based dissimilarities considering the total beta diversity and the turnover component, respectively. On the other hand, we did not find significant correlations when the environmental distance matrix was calculated using the set of environmental variables selected a priori. The results based on the bio-env analysis indicated, as expected, significant partial correlations between biological dissimilarities (independently of the component and facet) and environmental distances (Table 2). For the list of variables selected by bio-env, see Table S2.

**Table 2:** Partial Mantel correlation between biological dissimilarities and environmental distances (while controlling for spatial distances)

Variables	Facets	Components	*R*	*P*	CI-lower	CI-upper
All	Taxonomic	Total	**0.100**	0.041	0.05	0.15
All		Turnover	0.080	0.077	0.01	0.13
All		Nestedness	0.006	0.459	− 0.05	0.05
All	Traits	Total	0.130	0.075	0.007	0.20
All		Turnover	**0.120**	0.030	0.01	0.18
All		Nestedness	0.070	0.173	− 0.003	0.13
A priori	Taxonomic	Total	0.050	0.169	0.008	0.11
A priori		Turnover	0.016	0.393	− 0.03	0.07
A priori		Nestedness	0.040	0.257	− 0.05	0.09
A priori	Traits	Total	− 0.008	0.521	− 0.05	0.06
A priori		Turnover	0.060	0.112	0.03	0.10
A priori		Nestedness	− 0.050	0.736	0.03	0.10
Bio-env	Taxonomic	Total	**0.210**	0.001	0.16	0.26
Bio-env		Turnover	**0.200**	0.001	0.15	0.25
Bio-env		Nestedness	**0.140**	0.035	− 0.001	0.19
Bio-env	Traits	Total	**0.280**	0.008	0.17	0.35
Bio-env		Turnover	**0.220**	0.001	0.08	0.27
Bio-env		Nestedness	**0.220**	0.026	0.14	0.32

Analyses were run using the diatom data set and considering different methods to select environmental variables to calculate a single explanatory distance matrix (all variables, an a priori selected set of variables and variables selected by bio-env analysis), different facets of diversity (taxonomically-based and traits-based beta diversity), and components of beta diversity (total, turnover, nestedness-resultant) and CI = 95% confidence interval limit. Bold values indicate significant correlations. *P* values derived from bio-env should be interpreted with caution

### Macroinvertebrate taxonomically and traits-based beta diversity

Taxonomically-based beta diversity (mean total beta diversity = 0.481) was dominated by the turnover component (mean turnover component = 0.387), with a small contribution from nestedness (mean = 0.093) (Table 1). Also, we found low levels of traits-based beta diversity considering the three components (mean total beta diversity = 0.087, mean turnover = 0.032, and mean nestedness-resultant = 0.048; Table 1).

For taxonomically-based beta diversity, we found that the total and the turnover components were significantly correlated with the environmental distance matrix, either when this matrix was calculated with all environmental variables or with the a priori set of variables. For traits-based analyses, none of the strategies used to select variables produced environmental distances matrices significantly correlated with the beta diversity components. On the other hand, all beta diversity components (except nestedness-resultant dissimilarities for the species-based analysis) and facets were significantly correlated with the environmental distances when the bio-env analysis was used to select environmental variables (Table 3). For the list of variables selected by bio-env, see Table S3.

**Table 3:** Partial Mantel correlation results between biological dissimilarities and environmental distances (while controlling for spatial distances)

Variables	Facets	Components	*r*	*P*	CI-lower	CI-upper
All	Taxonomic	Total	**0.326**	0.001	0.27	0.37
All		Turnover	**0.321**	0.001	0.27	0.36
All		Nestedness	− 0.076	0.851	− 0.13	− 0.02
All	Traits	Total	0.079	0.151	− 0.03	0.13
All		Turnover	0.073	0.144	0.01	0.13
All		Nestedness	0.033	0.316	− 0.06	0.09
A priori	Taxonomic	Total	**0.242**	0.002	0.18	0.29
A priori		Turnover	**0.177**	0.004	0.12	0.23
A priori		Nestedness	0.045	0.213	− 0.01	0.07
A priori	Traits	Total	0.091	0.123	0.01	0.15
A priori		Turnover	0.024	0.372	− 0.03	0.08
A priori		Nestedness	0.094	0.106	0.02	0.15
Bio-env	Taxonomic	Total	**0.453**	0.001	0.40	0.49
Bio-env		Turnover	**0.431**	0.001	0.38	0.47
Bio-env		Nestedness	0.116	0.073	0.03	0.17
Bio-env	Traits	Total	**0.329**	0.003	0.11	0.40
Bio-env		Turnover	**0.243**	0.001	0.17	0.30
Bio-env		Nestedness	**0.297**	0.008	0.09	0.39

Analyses were run using the macroinvertebrate dataset and considering different methods to select environmental variables to calculate a single explanatory distance matrix (all variables, an a priori selected set of variables, and variables selected by bio-env analysis), different facets of diversity (taxonomically-based and traits-based beta diversity), and components of beta diversity (total, turnover, nestedness-resultant) and CI = 95% confidence interval limit. Bold values indicate significant correlations. *P* values derived from bio-env should be interpreted with caution

## Discussion

We found low correlations between the taxonomically-based and traits-based dissimilarities matrices (i.e., different facets of beta diversity) for both diatoms and macroinvertebrates and beta diversity components (turnover and nestedness). High values of correlation would indicate low dimensionality of beta diversity facets and, from this pattern, one could infer the role of a few underlying mechanisms. Our results point in the opposite direction and suggest that different mechanisms could explain the variation in each facet of beta diversity (for a discussion about dimensionality of biodiversity considering site-level measures, see Stevens and Tello 2014, 2018). In general, we can assert that taxonomically-based and traits-based beta diversity metrics offer complementary information, as has been found in other studies conducted in different types of ecosystems and using data of different groups of organisms (Gianuca et al. 2018; Heino and Tolonen 2017; Hill et al. 2019).

Diatom and macroinvertebrate taxonomically-based beta diversity varied substantially, whereas the variation of traits-based beta diversity was much smaller. The low values of traits-based beta diversity may result from functional convergence (i.e., adaptation of different species to similar habitat conditions; Villéger et al. 2013). Thus, different species occurring in different streams sites are sharing the same traits, leading to a low functional differentiation between stream sites (Statzner et al. 2004; Heino and Tolonen 2017). In general, our results are similar to those obtained by Villéger et al. (2013), who also found that, when compared to taxonomically-based beta diversity, lower levels of traits-based beta diversity were due to much lower turnover component and similarly low nestedness-resultant components. However, this topic has not been studied extensively from the traits-based perspective to date (Weinstein et al. 2014; Si et al. 2016; Hill et al. 2019), so making similar broad syntheses as for taxonomic beta diversity components (Soininen et al. 2018) is not yet possible.

By partitioning taxonomically-based and traits-based beta diversity using Baselga’s (2010) approach, we found similar patterns for diatoms and macroinvertebrates. In general, taxonomically-based beta diversity was largely driven by the turnover component, whereas the contribution of the nestedness component was minor. This pattern is consistent with the results of a recent meta-analysis, showing that the turnover component was, on average, 5.7 times larger than the nestedness component (Soininen et al. 2018). However, the nestedness component was slightly more important than the turnover component in the traits-based analyses. One reason for this result may be that, due to environmental filtering, some traits are more common than others, resulting in low traits-diversity assemblages being subsets of the high traits-diversity assemblages (Heino and Tolonen 2017). A similar result would occur if both environmental conditions and trait compositions vary in a nested way, resulting in a correlation between traits-based nestedness and environmental variation across a set of sites. For example, one could imagine that habitat heterogeneity ranges from very low to very high across sites. If so, then communities having a small set of traits would occur in the low heterogeneity sites and communities with a large variety of traits would occur in the high heterogeneity sites.

Ecologists have shown increasing interest in investigating the trait composition of biological communities, owing to the idea that traits might better reflect the responses of communities to environmental gradients than species identities (Poff 1997; McGill et al. 2006; Petchey and Gaston 2006; Verberk et al. 2013). Strong support for this idea would indicate that traits-based results are generalizable independently of taxonomic composition (Poff et al. 2006; Shipley et al. 2016). However, considering traits-based analyses, we found a significant correlation only for the turnover component of diatoms when all environmental variables were used to calculate the environmental distances (in addition to the results provided by the bio-env analysis). In addition, considering the overlaps of the confidence intervals (for the results based on taxonomic and traits data), we did not find evidence supporting that traits-based dissimilarities were more strongly correlated with environmental distances than with taxonomically-based dissimilarities. Even so, considering the high level of functional convergence (Table 1) in streams with naturally high levels of environmental heterogeneity (Table S1), the significant correlation between traits-based beta diversity and environmental distances is an interesting result. Diatoms are particularly responsive to environmental variation due to their small sizes and fast growth rates (e.g., Finlay and Fenchel 2004; Soininen et al. 2016). For instance, high-profile species are sensitive to physical disturbances and are not well adapted to unstable substrates (e.g., Passy 2007), whereas low-profile and motile diatoms are sensitive to nutrient enrichment (e.g., Passy 2007).

For macroinvertebrates, taxonomically-based beta diversity responded more strongly to environmental gradients than did traits-based beta diversity. A previous study on stream macroinvertebrates found that correlations between biological matrices and environmental conditions were lower for traits-based than for taxonomically-based analyses (Mueller et al. 2013). It is thus possible that diatom traits are more sensitive to environmental variation than those of macroinvertebrates, even though we selected traits for diatoms and macroinvertebrates that were as similar across these two groups as possible. One could argue that we focused on macroinvertebrate traits that are not strongly responsive to environmental conditions (Heino and Tolonen 2017), although we are confident that we used traits that should be associated with environmental variation as has been shown in previous studies on northern streams (Heino et al. 2007). However, depending on the ecological settings of a study area, some other traits may show little variation across sites and may not be responsive to environmental variation. For example, one can consider voltinism that varies only very little in the present study area (i.e., the majority of species have only one generation per year, whereas very few species are semivoltine). In such cases, including a trait that shows little variation across sites would only increase noise in the traits-based beta diversity data. Alternatively, because traits can be plastic within species, the traits possessed by macroinvertebrates in our study streams may differ from those for the same species elsewhere. For example, the macroinvertebrate trait data which we used come mostly from Central European literature, and given possible plasticity in the traits of the same taxa in climatically and environmentally different regions, these traits may not work in the best possible way in our near-pristine streams at high latitudes. However, we consider both explanations unlikely because a previous study on high-latitude streams has shown relatively strong relationships between traits and environmental variables (Tolonen et al. 2016). Finally, this result may also be related to the specific set of environmental variables which we used in our study.

The issue of variable selection in studies aiming to find correlates of biodiversity facets or metrics (e.g., richness, diversity, and beta diversity either adopting a taxonomic or a functional approach) is far from being trivial. For a raw data approach in the context of community analysis, there is a good solution in terms of acceptable type I and II error rates (e.g., Blanchet et al. 2008). Surprisingly, we lack a comparable and statistically sound solution when the response variable is a dissimilarity (beta diversity) matrix. For example, Ferrier et al. (2007) briefly discussed automated selection strategies, although these strategies have also been criticized (e.g., Whittingham et al. 2006). The application of information theoretic and Bayesian principles to multiple regressions on distance matrices has also been heavily criticized (Franckowiak et al. 2017). Thus, we used three approaches to select environmental variables and create a single, composite explanatory matrix. Our results derived from the selection of variables considering evidence from previous studies that highlighted the strong context dependency in stream ecology, although this approach proved to be poor in terms of finding significant correlates of beta diversity (see Heino et al. 2012; Tonkin et al. 2016). Similar results were obtained when all variables were selected for the calculation of the environmental distance matrix.

The search for strong environmental correlates is pivotal to test a basic assumption that favors traits-based ecology: “functional traits show general predictive relationships to measurable environmental gradients” (Shipley et al. 2016). Without a systematic test of a set of environmental variables, we would be unable to verify one of the promised main strengths of an ecological traits-based approach, namely, the capacity of “generalized prediction across organizational and spatial scales, independent of taxonomy” (Shipley et al. 2016). Thus, although significance levels from the bio-env analysis cannot be trusted (due to the maximization of the correlation between matrices), this analysis provided us with a list of variables that is worth to be tested in future studies using independent datasets. For diatoms, our results indicate that substrate characteristics (e.g., sand, gravel, and pebble), conductivity, and stream width are likely to be important in explaining traits-based beta diversity, whereas the list includes nitrogen concentration, pH, depth, and shading for macroinvertebrates. However, we caution that these lists are likely to perform poorly in distant geographical regions because of context dependency imposed by environmental and biological differences among drainage basins.

Similarly, without testing the same set of environmental variables in different systems, we would also be unable to evaluate the pervasiveness of context dependence in ecological systems (different results would be simply explained by the use of different variables). For example, in the context of selecting variables for future studies, those indicated by bio-env for taxonomically-based beta diversity of both groups of organisms (i.e., boulder, moss, depth, and shading) have, indeed, been found to be important for different stream communities (Heino and de Mendoza 2016; Jyrkänkallio-Mikkola et al. 2017). Among these variables, we highlight the role of shading, which, mediated by its effects on light availability and temperature, is related to different in-stream processes (e.g., periphyton biomass accrual; Mosisch et al. 2001) and, ultimately, to community assembly (e.g., McCall et al. 2017). Using bio-env, we also found that moss cover was important for community dissimilarities. Moss cover is generally important for stream macroinvertebrate distributions (Hildrew 1977; Heino and Korsu 2008), although the response may be either indirect (e.g., a species uses moss as a temporary habitat), direct (i.e., a species cannot construct filtering nets without moss cover), or resource-based (i.e., a species feeds on moss). Finally, the difference between the variables selected in traits- and taxonomically-based analyses are in line with what would be expected due to the low relationship between these facets (i.e., low correlations between the taxonomically-based and traits-based dissimilarities matrices).

In conclusion, when all environmental variables were used to calculate the explanatory matrix, we found a significant correlation between taxonomically-based dissimilarities, for both diatoms and macroinvertebrates, and environmental distances (after accounting for spatial distances). Limiting the discussion to statistically significant results, this result was the unique consistent finding across organism groups, facets, components, and approaches to select environmental variables. In other words, the results diverged for other comparisons (e.g., the turnover component of the traits-based matrix of beta diversity was significantly correlated with the environmental distance matrix for diatoms only). However, the significant relationship between beta diversity and environmental distances provides support for the role of environmental filtering as a driver of community dissimilarities of rather different biological groups. This finding is interesting especially considering the pristine nature of our stream sites. Thus, in an applied context and when analyzed over broad human-induced environmental gradients, beta diversity would likely to be even more indicative of environmental effects on biological communities. For different facets and components of beta diversity, we did not find an increase in the explanatory power when the variables were selected based on previous works on macroinvertebrates (e.g., Tolonen et al. 2016) and diatoms (e.g. Lindholm et al. 2018), highlighting the context dependence in community–environment relationships (Heino et al. 2012, Alahuhta and Heino 2013). We only found support for the view that traits-based approaches have the potential to increase generality and predictability in community ecology (Poff 1997; Poff et al. 2006; McGill et al. 2006) when variables were selected via the bio-env analysis, which was mainly true for diatoms. Therefore, we believe that both taxonomically and traits-based approaches are still needed to better understand the mechanisms underlying beta diversity patterns.

## Electronic supplementary material

Below is the link to the electronic supplementary material.

Supplementary material 1 (DOCX 817 kb)
