# Supplementary material for: Correlates of different facets and components of beta diversity in stream organisms
Source: Oecologia. 2019 Oct 17;191(4):919–29. doi: 10.1007/s00442-019-04535-5 (PMC6853853; doi:10.1007/s00442-019-04535-5)
Supplement: Supplementary file 1 — Supplementary material 1 (DOCX 817 kb) [file 442_2019_4535_MOESM1_ESM.docx]

*Supporting Information*

Oecologia

**Correlates of different facets and components of beta diversity in stream organisms**

Mariana Perez Rocha^1,2,*^, Luis M. Bini^3^, Mira Grönroos^4^, Jan Hjort^1^, Marja Lindholm^1^, Satu-Maaria Karjalainen^2^, Katri E. Tolonen^2^ & Jani Heino^2^

^1^University of Oulu, Geography Research Unit, P.O. Box 3000, FI-90014 Oulu, Finland

^2^Finnish Environment Institute, Freshwater Centre, P.O. Box 413, FI-90014 Oulu, Finland

^3^Department of Ecology (ICB), Universidade Federal de Goiás, Goiânia, GO, 74690-900, Brazil

^4^Faculty of Biological and Environmental Sciences Ecosystems and Environment Research, Niemenkatu 73, 15140 Lahti, Finland

*CAPES Foundation, Ministry of Education of Brazil, Brasília - DF 70040-020, Brazil

Correspondence to: [mperezrocha@gmail.com](mailto:mperezrocha@gmail.com)

**Table S1.** Descriptive statistics of all environmental variables used to estimate environmental distances. Min. = minimum values, Max. = maximum values, CV = coefficient of variation.

| Variables | Min. | Max. | CV (%) |
| --- | --- | --- | --- |
| Total Nitrogen (μg/L^-1^) | 62 | 260 | 34.27 |
| Color (pt-Co) | 10 | 50 | 35.20 |
| Iron (μg/L^-1^) | 8 | 160 | 61.15 |
| Manganese (μg/L^-1^) | 1 | 5.5 | 69.66 |
| pH | 6.58 | 7.51 | 2.46 |
| Conductivity (μS/cm^-1^) | 16 | 36 | 20.14 |
| Sand (mm) | 0 | 24.5 | 400.16 |
| Gravel (mm) | 0 | 12 | 113.60 |
| Pebble (mm) | 0 | 45.67 | 76.92 |
| Cobble (mm) | 1 | 52 | 48.83 |
| Boulder (mm) | 7.08 | 99 | 38.78 |
| Moss (%) | 0.3 | 75 | 111.04 |
| Velocity (m/s^-1^) | 0.28 | 0.89 | 22.43 |
| Depth (cm) | 14.6 | 34.47 | 19.45 |
| Stream width (m) | 1.2 | 22 | 72.01 |
| Shading (%) | 0 | 100 | 78.29 |

**Table S2**. Variables selected by bio-env for diatom taxonomically-based (A) and traits-based data (B).

|  | **(A)Taxonomic** | | | **(B) Traits** | | |
| --- | --- | --- | --- | --- | --- | --- |
| Variables | Total | Turnover | Nestedness | Total | Turnover | Nestedness |
| Nitrogen |  |  |  |  | x |  |
| Manganese | x | x |  |  |  |  |
| Color |  |  | x |  |  |  |
| Conductivity | x | x |  | x |  |  |
| Sand |  |  |  | x | x | x |
| Gravel |  |  |  | x |  | x |
| Pebble |  |  |  | x |  | x |
| Boulder | x | x |  |  |  |  |
| Moss | x | x |  |  |  |  |
| Current velocity | x | x |  |  |  |  |
| Depth | x | x |  |  |  |  |
| Stream width |  |  | x | x |  | x |
| Shading | x | x |  |  | x |  |

**Table S3**. Variables selected by bio-env for macroinvertebrate taxonomically-based (A) and traits-based data (B).

|  | **(A)Taxonomic** | | | **(B)Traits** | | |
| --- | --- | --- | --- | --- | --- | --- |
| Variables | Total | Turnover | Nestedness | Total | Turnover | Nestedness |
| Nitrogen | x | x |  | x |  | x |
| Iron |  | x |  |  |  |  |
| pH |  |  | x | x | x | x |
| Boulder | x | x |  |  |  |  |
| Moss | x | x |  |  | x |  |
| Depth | x | x |  | x | x |  |
| Shading | x | x |  |  | x |  |
| Color |  |  | x |  |  |  |

**Table S4**. Results of Mantel tests ran separately within each organismal group, diatoms and macroinvertebrates, correlating taxonomically-based and traits-based dissimilarity matrices (total, turnover, and nestedness-resultant).

| **Diatoms** |  |  |  |  | **Macroinvertebrates** |  |  |  |  |
| --- | --- | --- | --- | --- | --- | --- | --- | --- | --- |
| Taxonomic/Trait  dissimilarities | *r* | *P* |  |  | Taxonomic/Trait  dissimilarities | *r* | *P* |  |  |
| Total | 0.263 | 0.001 |  |  | Total | 0.512 | 0.001 |  |  |
| Turnover | 0.231 | 0.001 |  |  | Turnover | 0.366 | 0.001 |  |  |
| Nestedness | 0.249 | 0.002 |  |  | Nestedness | 0.445 | 0.001 |  |  |


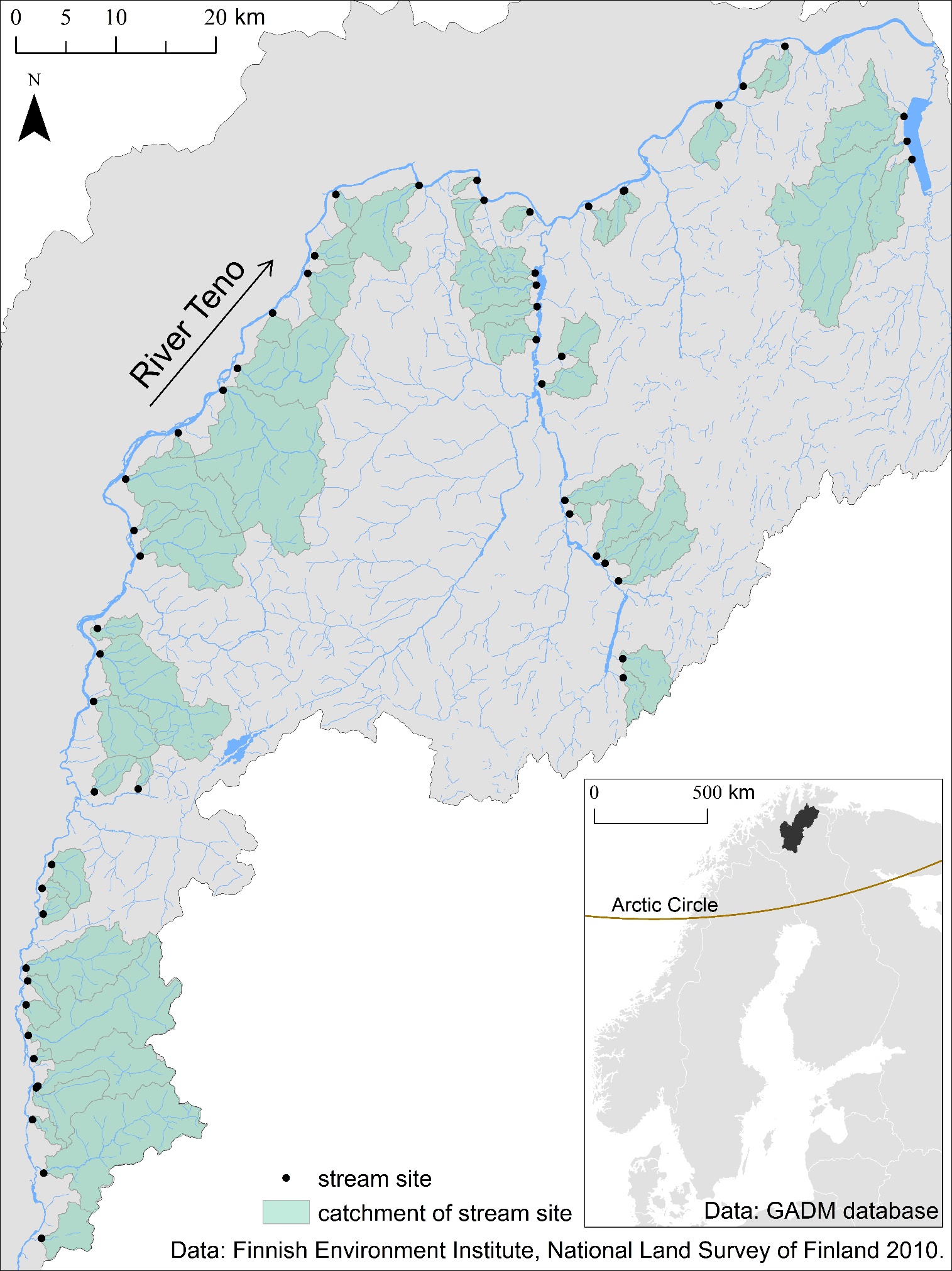


**Figure S1**. Map of the study area: the Tenojoki River basin. Black full dots denote the location of sampled points (54 streams sites). Green color shows the catchment of each stream.


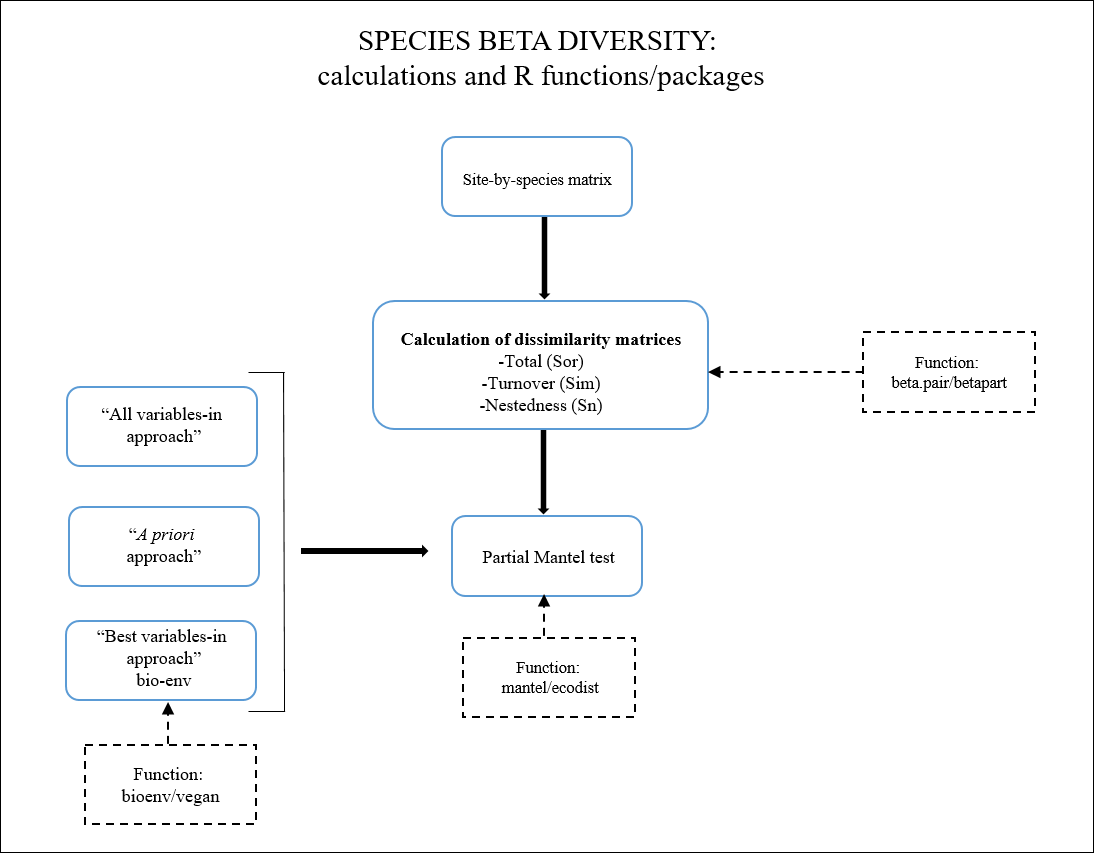


**Figure S2**. A flow-chart of taxonomic-level beta diversity analyses used for diatom and macroinvertebrate data. Sor = Sørensen; Sim = Simpson; Sn = nestedness-resultant component.


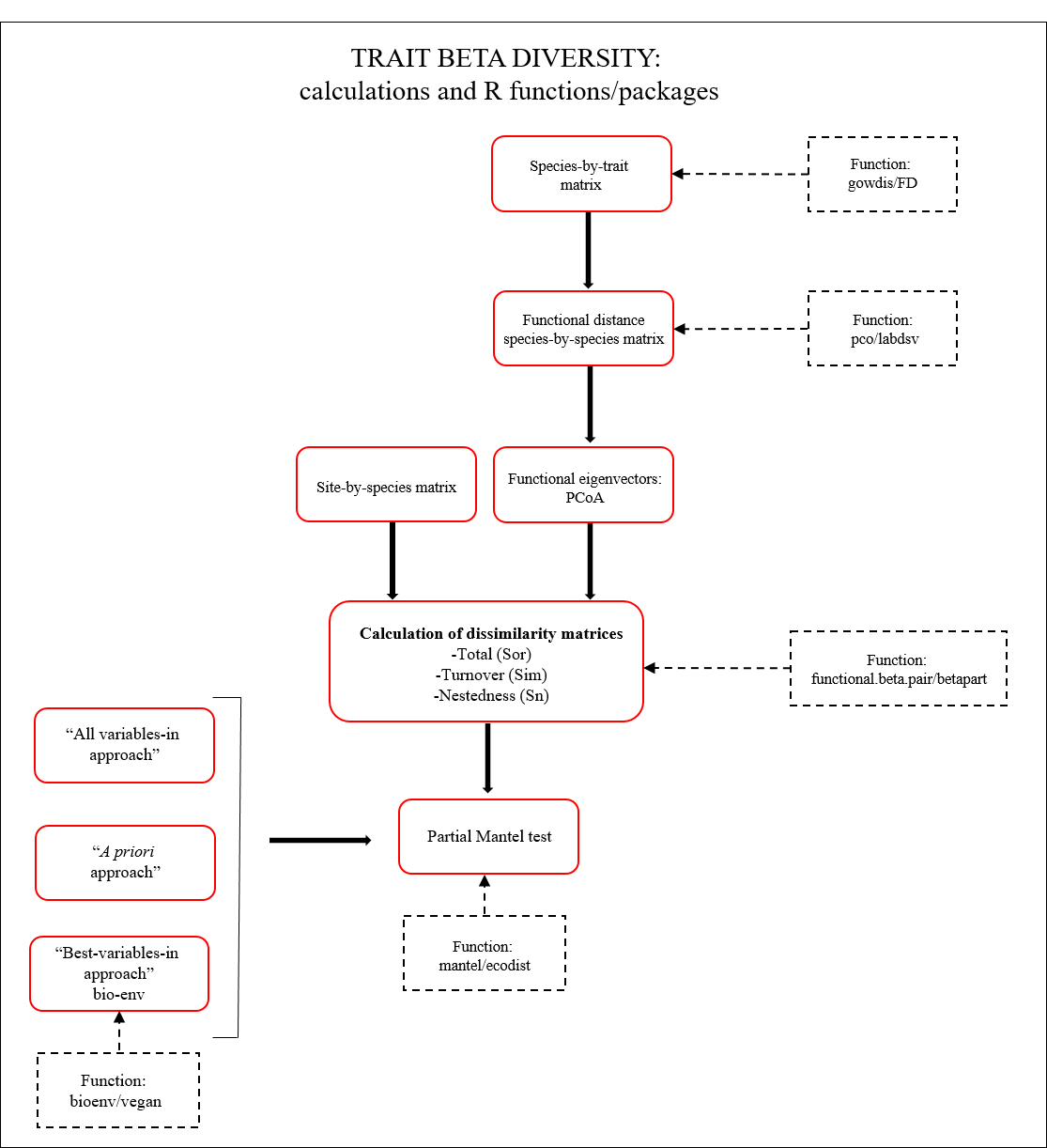


**Figure S3**. A flow-chart of traits-level (functional) beta diversity analyses used for diatom and macroinvertebrate data. Sor = Sørensen; Sim = Simpson; Sn = nestedness-resultant component.
